# General Morphology and Ultrastructure of the Venom Apparatus and Convoluted Gland of the Fire Ant, *Solenopsis saevissima*

**DOI:** 10.1673/031.010.2401

**Published:** 2010-03-23

**Authors:** Eduardo Gonçalves Paterson Fox, Odair Correa Bueno, Antônio Teruyoshi Yabuki, Carlos Massuretti de jesus, Daniel Russ Solis, Mônica Lanzoni Rossi, Neusa de Lima Nogueira

**Affiliations:** ^1^ Centro de Estudos de Insetos Sociais, UNESP, Rio Claro — SP; ^2^ Laboratório de Histopatologia e Biologia Estrutural de Plantas, USP, Piracicaba — SP

**Keywords:** Solenopsidini, internal anatomy, scanning electron microscopy, transmission electron microscopy

## Abstract

A group of 13 species of the genus *Solenopsis* is markedly difficult to assess taxonomically, although they are of considerable economical and medical importance in some countries where some of them were introduced. These ants are aggressive and their venomous stings can be very allergenic. The venom apparatus has been described in fine detail for only two of these species, and differences in this structure among the different species might prove useful as taxonomic characters. The venom apparatus of *Solenopsis saevissima* Smith (Hymenoptera: Formicidae) is herein described with the aid of light and electron microscopy techniques, and compared to that of *S. invicta* and *S. richteri.* The cellular organization of the different parts present differences that suggest functional specialization. In general, the different tissues were abundant in vesiculae and mitochondria, but presented little endoplasmic reticulum and few ribosomes, probably because they produce little protein. The length of the free filaments of the venom gland and the width of their internal ducts seems to vary from what was described for *S. richteri,* but this may be of little use to taxonomy.

## Introduction

Venom apparatuses are common structures of hymenopterans and are involved in the production of active compounds to be delivered through an ovipositor or sting. Many hymenopterans have stings, which, apart from being used to subdue their prey, can be used effectively for defense. In some ants, the sting is used for colony defense, and some people can develop serious anaphylactic reactions to ant venoms (Brown and Heddle 2003).

Some ants of the genus *Solenopsis* Westwood (Hymenoptera: Formicidae) are known as fire ants (Vinson 1986) because of their painful stings. They aggressively attack in swarms when their fragile, earthen nests are disturbed. Fire ants are native to the Americas and most diverse in South America, but some species of this group have been shipped and introduced into other world regions inadvertently. At least one species, *Solenopsis invicta* Buren, has become a major public concern, mainly in the United States, because of its marked adaptability to human environments and the allergenicity of its sting (Rhoades et al. 1989; deShazo and Banks 1994; deShazo and Williams 1995; deShazo et al. 1999; Kemp et al. 2000). One species, *Solenopsis saevissima* Smith, is still restricted to South America and common in Brazil (Rossi and Fowler 2004). It has not been studied as extensively as *S. invicta.*

Both species belong to a particularly problematic ant group, in terms of taxonomy and systematic, known as the “*Solenopsis saevissima* group of species” (Pitts et al. 2005). It includes 13 fire ant species that exhibit marked morphological similarity and intraspecific variability. Some species are capable of hybridization, rendering most morphological characters for species separation unreliable (Vander Meer 1985; Pitts et al. 2005). There is still some ongoing discussion about the validity of these species and the best characters to be used in defining each species (Ross and Trager 1990; Ross and Shoemaker 2005).

The venom apparatus of *Solenopsis richteri* was thoroughly described, including histological aspects, by Callahan et al. (1959). Later, the venom apparatus of *S. invicta,* a similar species with which *S. richteri* can hybridize (Vander Meer 1985), was briefly described by Billen (1990), who also analyzed some ultrastructural aspects of it. No other venom apparatuses of any species of this group have been described, but it is well known that the venoms of the different species of fire ants have distinct chemical composition (Jones and Blum 1982; Fox, Palma and Bueno, unpublished data). The different compositions might reflect differences in the internal organization of the structures of the venom apparatus, and some of these differences might help elucidate the systematics for this group.

The present investigation about the morphological and cellular organization of the venom apparatus of *S. saevissima* was carried out, pointing out specific differences through comparison of the observed structures with what has been done with other species in the genus.

## Materials and Methods

The ants were obtained from a house garden in the outskirts of Pedro do Rio, RJ (22°20′30.45″S; 43°07′44.51″W), following the methods for collecting, handling and rearing fire ants in the laboratory as described by Banks et al. (1981).

The venom apparatuses were dissected under a stereomicroscope with fine tweezers from cold-anesthetized ants into a droplet of 0.09% saline solution and were transferred into an eppendorf tube with Dietrich's solution (900 ml distilled water, 450 ml 95% ethanol, 150 ml 40% formaldehyde, 30 ml acetic acid). Some venom apparatuses were dissected and placed in a droplet of saline to be analyzed directly under a stereomicroscope without fixing. Digital pictures of these were taken with a Sony Cybershot digital camera directly attached to the ocular lens. The following procedures were completed about 24h later.

## Samples for optical microscopy

Ten venom apparatuses were dehydrated with a graded ethanol series and placed in paraffin blocks, which were cut into 7 µm sections and later stained with haematoxylin and eosin for analysis under an optical microscope (Zeiss Axiostar, www.zeiss.com). Digital pictures of the cuts were taken with a Sony Cybershot digital camera directly placed over an ocular lens.

## Samples for scanning electronic microscopy (SEM)

Ten venom apparatuses were rinsed thrice with 0.1 M sodium cacodylate buffer (pH 7.2), post-fixed with 1.0% osmium tetroxide for one hour and dehydrated in a graded series of ethanol, then submitted to critical-point drying with CO2. After this, the dried samples were mounted over aluminium stubs with double-faced adhesive tape and gold-covered with a Balzers MED 010 ‘sputterer’ device. These were analyzed under the Zeiss LEO 435 VP microscope at 20 kv as soon as possible.

## Samples for transmission electronic microscopy (TEM)

Some Ten venom apparatuses were rinsed thrice with 0.1 M sodium cacodylate buffer (pH 7.2), post-fixed with 1.0% osmium tetroxide for two hours, and then dehydrated in a graded acetone series, embedded in ‘Spur’ resin. Once solidified, these blocks were cut alternately with a microtome in 120 nm / 60–90 nm-thick sections. The semi-thin sections were mounted over glass slides and stained by briefly heating with toluidine blue, while the thinner sections were mounted over prepared copper grids and stained with 2.5% uranyle acetate (40 min) and lead citrate (20 min) (Reynolds 1963). The semi-thin sections were used for locating the areas of interest in the blocks, and then thin sections were taken and observed under a Zeiss EM-900 electron microscope at 50 kv.

## Results

**Figure 1.**
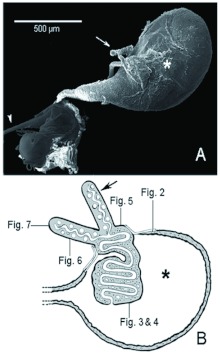
General organization of the venom apparatus of *Solenopsis saevissima.* A) External morphology of the venom apparatus through SEM. B) Schematic representation of a wholly sectioned venom gland with blueprints to figure of each region. On both illustrations: * = venom sac; arrows = free filaments; arrowhead = sting. In the scheme: cellular nuclei in varied forms are represented as white spheres; trachea are represented as tubes near the free filaments; mitochondria are represented as black dots. High quality figures are available online.

The venom apparatus of *S. saevissima* was a sac-like reservoir with two tubular filaments located at the distal end of the gaster (Figure 1A). The whitish venom reservoir (about 754 µm long × 362 µ m wide) was slightly transparent with a rugous surface. The convoluted gland had a faint yellowish hue that could be seen in the interior. The free filaments were delicate, semi-transparent and about 435 µm long (Figure 1A). The basal end of each filament was attached to the reservoir, and the apical end was situated freely in the body cavity. The free filaments were internally continuous with the convoluted gland (Figure 1B).

**Figure 2.**
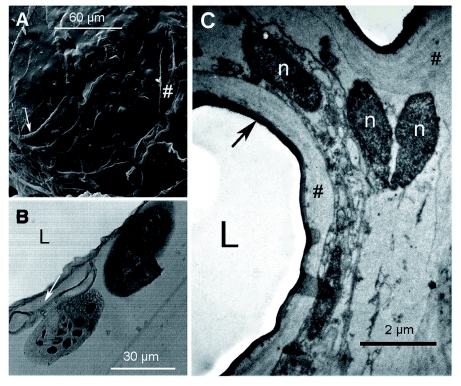
Venom reservoir wall of *Solenopsis saevissima.* A) SEM detail on the surface; arrow = associated trachea; # = and ruptures on the wall. B) Optical image of a cross section of the reservoir and filaments; white arrow = associated tracheae. C) Fine structure of the reservoir wall; black arrow = cuticle; n = cellular nuclei; # = irregular tunica propria. High quality figures are available online.

At the base of the filaments on the venom reservoir, there were abundant intruding trachea (Figure 1B, 2A). The ultrastructure of the reservoir wall is shown in Figure 2B. The ultrastructure consisted of a soft tissue of sparse irregular cells with small ovoid nuclei, some endoplasmic reticulum, and a few vesicles. This tissue was surrounded on both sides by a tunica propria of variable width completely lined with a continuous 1 µm-thick cuticle (Figure 2B). In Figure 2A, the outer cuticle has been torn in some regions during the processing of the sample, revealing the rugous surface of the tunica propria lying underneath.

**Figure 3.**
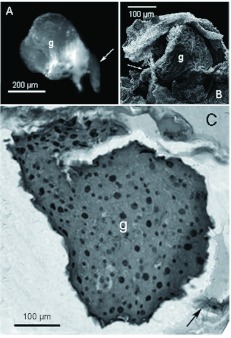
Convoluted gland of *Solenopsis saevissima.* A) Light microscopy micrograph of a dissected convoluted gland; arrow = translucent intermediary zone; g = internal zone. B) SEM image; g = convoluted gland inside a ripped reservoir. C) Light microscopy micrograph of a transverse section of the convoluted gland (g) in the reservoir; arrow = exit duct. High quality figures are available online.

The convoluted gland was a delicate, semi-transparent, yellowish mass inside the venom reservoir. Interestingly, when some portion of the gland was gently pulled with a fine forceps, it continuously uncurled as a long, apparently unbranched, sinuous, semi-transparent thread (not shown). In Figure 3A, it has been completely removed from the venom reservoir, showing its irregular surface that was more transparent and delicate at the base of the free filaments. This particular region will be here referred to as the “intermediary zone.”

The convoluted gland was roughly shaped like a brain and occupied much of the internal volume of the venom reservoir (Figure 3B, Figure 3C). The convoluted gland is a prolongation of the free filaments.

**Figure 4.**
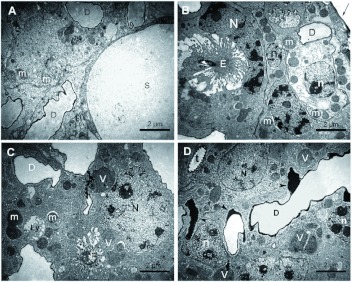
Fine structure of the convoluted gland of *Solenopsis saevissima.* In all images: S = vesicle with secretion; N = nucleus of secretory cell; n = nucleus of duct cell; v = vesicle; D = duct; Ly = lysosome; m = mitochondrion; E = end apparatus; t = tracheole; black arrow = black cuticle; arrowheads = ducts containing electron-dense material inside. High quality figures are available online.

Through ultrastructure, the convoluted gland was composed of an intertwined mass of class III gland cells (cell complexes described by Noirot and Quennedey (1974) as bicellular units of closely associated secretory and duct cells), sinuous internal ducts, and tracheoles of various diameters (Figure 4). It was also lined with a continuous dark cuticle, and there were big vesicles with secretion (Figure 4A, B). It was difficult to discern between the two cell types of the cell complex because they were similar and the limits were irregular, but the duct cells were typically abundant in mitochondria, and irregularly shaped with roughly spherical nuclei ranging 1–3 µm in diameter (Figure 4B, C). The secretory cells were larger and more-regularly shaped, with nuclei of various shapes ranging 3– 8 µ m in size, often having markedly darker cytoplasm (Figure 4D). Both cell types frequently contained dark vesicles of various sizes (Figure 4C, D), within some of which traces of organelles could be seen (not shown), suggesting that some of these vesicles were some type of lysosome. Both cell types presented nuclei with different degrees of cromatin condensation, and they usually contained a few smaller vesicles and endoplasmic reticulum (not shown). Neither golgi complexes nor rugous enoplasmic reticula were observed. Inside the convoluted gland, duct cells were more abundant than secretory cells. Secretory cells presented end apparatuses (invaginated spaces lined with microvilli linking ductules to secretory gland cells as defined by Noirot and Quennedey (1974)) (Figure 4A, B, C). Tracheoles of various diameters were sporadically observed (Figure 4D), and the sinuous ducts (of irregular shape and calibres) were abundant in the convoluted gland (Figure 4C, D).

**Figure 5.**
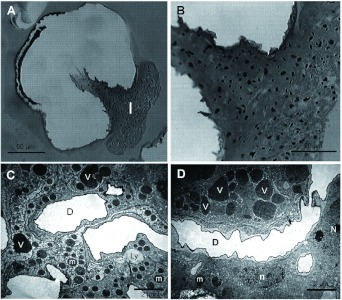
Intermediary zone of *Solenopsis saevissima.* A) Cross section of the venom reservoir, displaying the intermediary region between the convoluted gland and base of free filaments; I = intermediary zone. B) Closer view of the intermediary zone. C) and D) Fine structure aspects of the intermediary zone; D = duct; n = nucleus of duct cell; v = vesicle; m = mitochondrion; Ly = lysosome. High quality figures are available online.

Some ducts had electro-dense material inside (Figure 4D).

The intermediary zone was the delicate semitransparent zone between the convoluted gland and the free filaments; it was positioned externally to the venom reservoir, and it was generally similar in cellular organization to the convoluted gland (compare Figure 3C with Figure 5A, B). In this intermediary region, the ducts were much more abundant, but neither end apparatuses nor tracheoles were observed. This suggests that it is mainly composed of duct cells. The duct cells of this region were markedly abundant in mitochondria and dark vesicula, which tended to form clusters (Figure 5C, D). Myellinic bodies in the cells were occasionally seen (not shown) and some lysosomes were observed (Figure 5C).

**Figure 6.**
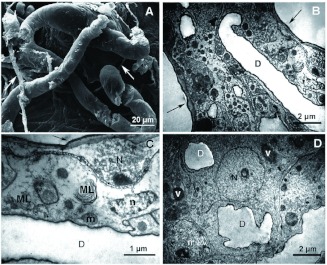
Proximal region of free filaments of *Solenopsis saevissima.* A) External SEM image of the free filaments; arrows = associated tracheae. B) Fine structure of the proximal region of a free filament; D = central duct. C) Closer view on part of the previous image, showing a plasmolyzing cell; N = nucleus; D = central duct; ML = multilamellar inclusion; m = mitochondrion; n = duct cell nucleus. D) Ultrastructural closer view of another area in the same region; N = secretory cell nucleus; n = duct cell nucleus; D = duct; v = vesicle. High quality figures are available online.

**Figure 7.**
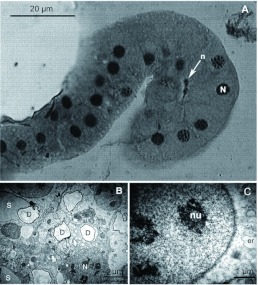
Distal region of free filaments of *Solenopsis saevissima.* A) Light microscopy micrograph of a transversal section of the tip of a filament, n = nucleus of duct cell; N = secretory cell nucleus. B) Fine structure of the distal section of a free filament; m = mitochondrion; N = secretory cell nucleus; D = duct; S = vesicle with secretion. C) Detail on a cellular nucleus; nu = nucleole; er = endoplasmic reticulum. High quality figures are available online.

The free filaments were of continuous width and had a smooth surface. They were also externally lined with a thin cuticle (Figure 6A, B). There was a gradual change of cellular organization from the intermediary zone to a more organized cubic epithelium surrounding a central collecting duct (Figure 6B). At the proximal region of the filaments, some mitochondria and vesicles were present inside the duct cells, and multilamellar inclusions (Figure 6C, D) and a few end apparatuses (not shown) were observed. Toward the distal portion of the filaments (Figure 7A) the cubic cells of the epithelium became gradually larger and more abundant. They had clearer cytoplasm, few small mitochondria and large round nuclei with well-defined borders (Figure 7B). Again, no ribosomes or golgi complexes were observed. Ducts were less abundant, and, consequently, few duct cells were observed (Figure 7A, B). No tracheoles or end apparatuses were found in this region. At the tip of the free filaments, these cubic cells were predominant. The detail of a nucleus of one of these cells is presented in Figure 7C, where a vesicle of endoplasmic reticulum can be seen.

## Discussion

The general aspect of the venom apparatus of this species is similar to what was described for *S. invicta* and *S. richteri* (Callahan et al. 1956; Billen 1990), but markedly different from those described for ants of other genera (Schoeters and Billen 1995; Ortiz and Camargo-Mathias 2003; Nunes and Camargo-Mathias 2005; Ortiz and Camargo-Mathias 2005). The lack of muscle fibers associated with the venom reservoir indicates that the propelling force for the venom to be injected must be provided by a strong contraction of the gaster. As a consequence, the venom reservoir would have to be a relatively resistant structure because of the soft internal tissue and tunica propria within the continuous outer cuticle.

The fact that the convoluted gland is formed by a single, greatly-coiled, long duct forming a mass inside the venom reservoir agrees with the description of some other ants by Schoeters and Billen (1998), but it is radically different from the proposed model of this gland as illustrated in Billen (1990). The proposed model in Billen (1990) suggests that the venom gland of *S. invicta* is strikingly different from that of *S. saevissima.* The convoluted glands in the *S. saevissima* specimens were never immersed completely in the venom reservoir, as was shown in Billen (1990), where the proposed model entirely lacked an external intermediary zone. Some glands of *S. invicta* were dissected and observed directly confirming that the general disposition of the apparatus was similar to that of *S. saevissima* and to what was described for *S. richteri* by Callahan et al. (1959). The convoluted gland was composed of a single, long convoluted tube, without the side ramifications of the collecting duct proposed by the model in Billen (1990).

There were differences between these results and the findings of Callahan et al. (1959). These authors repeatedly illustrated the convoluted gland inside the venom reservoir of *S. richteri* as roughly elliptical, while the shape of this gland in these sections resembled that of a brain or mushroom. In their illustrations of the venom gland, Callahan et al. (1959) described and illustrated, in detail, the internal organization of the various parts of the venom apparatus. The cellular disposition in the free filament cells was similar to the present observations, but the cellular nuclei in the free filaments of *S. saevissima* appeared to be much bigger than the nuclei of the secretory cells of the convoluted gland and intermediary zone. The drawings of *S. richteri* in Callahan et al. (1959) indicate the opposite. Moreover, the main collecting duct in the free filaments was represented in the drawings of Callahan et al. (1959) as a clear and continuous tube inside the free filaments, while the same duct inside the filaments of *S. saevissima* seemed markedly narrow and sinuous, even difficult to detect in some sections. Lastly, the free filaments of the venom gland of *S. richten* were much longer than those observed for *S. saevissima,* although they had roughly the same diameter. As these traits were repeatedly illustrated by Callahan et al. (1959), these differences should be directly verified. For this study, there were no readily obtainable *S. richten* workers. If these differences prove to be discernible among different fire ant species, they may be of some utility to systematics and taxonomy. It should be noted that cellular differences in size might reflect differences in physiological status; thus these should be considered with caution in comparative studies.

As mentioned by Billen (1990), the venom of these ants is composed generally of piperidine alkaloids (see also Brown and Heddle 2003) and has very low protein content. This was reflected in the absence of granular endoplasmic reticulum in the cells of the venom apparatus. Mitochondria, however, were abundant (Figures 4B, 5B, 6B; Billen 1990), as were vesicles (Figure 4A), thus indicating the intense production of compounds and metabolism within this organ.

The fine cellular structure of the venom apparatus and the distinct differences in tissue organization of the various parts, e.g. the intermediary zone and the free filaments, likely reflects specialization of the secretory activity of each region. Most of the passage of synthesized substances into the convoluted duct probably takes place inside the convoluted gland, where end apparatuses were markedly abundant. Most of the synthesis was observed in the intermediary zone, and some was observed in the convoluted gland. The tightly intertwined duct was described by Callahan et al. (1959) as presenting only one discharging exit to the venom reservoir. Therefore, some changes should occur with the collected products before entering the venom sac.

The semi-obstructed ducts observed may be correlated with the observations made by Callahan et al. (1959), in which the venom had crystallized inside the ducts in some regions, possibly clogging the final exit duct. The multilamellar inclusions observed (Figure 6C) could be correlated with the observations of Callahan et al. (1959) where some cells plasmolyzed in the venom gland, possibly as a consequence of this duct obstruction in the convoluted gland. This may have something to do with possible biochemical changes occurring inside the long duct. The multilamellar inclusions (Figure 6C) were found inside the duct cells, suggesting that such cells may be short lived, possibly because of the intensity and nature of their metabolic activities and the toxic nature of their secretions. Multilamellar inclusions were also observed previously by Billen (1991) in ant secretory glands and end apparatus, and the author suggested that those could be products of secretion, possibly in association with lipidic compounds. These inclusions may be correlated with the function of the long convoluted duct and possibly with extracellular alterations to the venom secretions, thus their true nature would credit deeper investigation.

The results suggest that the venom apparatus is composed of simple partitioned structures that produce different compounds. The composition of the electron-dense vesicles inside the duct cells of the convoluted gland and intermediary region is unclear, but some remains of cellular materials were noticed inside some of them (e.g. membranes), thus some could actually be lysosomes. Those vesicles probably do not carry venom secretions, because they are much more eletron-dense than the contents of the ducts, the venom reservoir and the end apparatuses. Additional histochemical studies are necessary to help understand those structures and more clearly elucidate the function of the apparatus as a whole.

The results suggest that most secretions are produced directly by the venom duct cells, especially those of the intermediary zone and those in the convoluted gland. There seems to be little metabolism in the free filaments, and no substances seem to be produced by the reservoir at all.

The differences (i.e., general aspect and length of the free filaments) observed between the venom apparatus of *S. saevissima* and that of the other fire ant species were only slight; therefore, they will likely be of little use in taxonomy.

## Abreviations

SEM: scanning electron microscopy;
TEM: transmission electron microscopy
